# Muscle ion transporters and antioxidative proteins have different adaptive potential in arm than in leg skeletal muscle with exercise training

**DOI:** 10.14814/phy2.13470

**Published:** 2017-10-16

**Authors:** Magni Mohr, Tobias Schmidt Nielsen, Pál Weihe, Jákup A. Thomsen, Giovanna Aquino, Peter Krustrup, Nikolai B. Nordsborg

**Affiliations:** ^1^ Centre of Health Sciences Faculty of Health Science University of the Faroe Islands Tórshavn Faroe Islands; ^2^ Center for Health and Human Performance Department of Food and Nutrition, and Sport Science University of Gothenburg Gothenburg Sweden; ^3^ Department of Nutrition, Exercise and Sports Section of Human Physiology University of Copenhagen Copenhagen Denmark; ^4^ Department of Occupational Medicine and Public Health The Faroese Hospital System Tórshavn Faroe Islands; ^5^ Department of Movement Sciences and Wellness (DiSMEB) University “Parthenope” Naples Italy; ^6^ CEINGE‐Advanced Biotechnologies Naples Italy; ^7^ Department of Sports Science and Clinical Biomechanics Faculty of Health Sciences SDU Sport and Health Sciences Cluster (SHSC) University of Southern Denmark Odense Denmark; ^8^ Sport and Health Sciences College of Life and Environmental Sciences University of Exeter Exeter United Kingdom

**Keywords:** Acid/base regulation, antioxidant capacity, soccer, sodium/potassium pump, swimming

## Abstract

It was evaluated whether upper‐body compared to lower‐body musculature exhibits a different phenotype in relation to capacity for handling reactive oxygen species (ROS), H^+^, La^−^, Na^+^, K^+^ and also whether it differs in adaptive potential to exercise training. Eighty‐three sedentary premenopausal women aged 45 ± 6 years (mean ± SD) were randomized into a high‐intensity intermittent swimming group (HIS,* n *=* *21), a moderate‐intensity swimming group (MOS,* n *=* *21), a soccer group (SOC,* n *=* *21), or a control group (CON,* n *=* *20). Intervention groups completed three weekly training sessions for 15 weeks, and pre‐ and postintervention biopsies were obtained from deltoideus and vastus lateralis muscle. Before training, monocarboxylate transporter 4 (MCT4), Na^+^/K^+^ pump *α*_2_, and superoxide dismutase 2 (SOD2) expressions were lower (*P *<* *0.05) in *m*. deltoideus than in *m*. vastus lateralis, whereas deltoid had higher (*P *<* *0.05) Na^+^/H^+^ exchanger 1 (NHE1) expression. As a result of training, Na^+^/K^+^ pump *α*_2_ isoform expression was elevated only in deltoideus muscle, while upregulation (*P *<* *0.05) of the *α*_1_ and *β*_1_ subunits, phospholemman (FXYD1), NHE1, and superoxide dismutase 1 expression occurred exclusively in vastus lateralis muscle. The increased (*P *<* *0.05) expression of MCT4 and SOD2 in deltoid muscle after HIS and vastus lateralis muscle after SOC were similar. In conclusion, arm musculature displays lower basal ROS, La^−^, K^+^ handling capability but higher Na^+^‐dependent H^+^ extrusion capacity than leg musculature. Training‐induced changes in the ion‐transporting and antioxidant proteins clearly differed between muscle groups.

## Introduction

The majority of research focusing on human skeletal muscle adaptation to exercise training investigates only *m*. vastus lateralis, and the fact that leg musculature may not be representative of all muscle groups is often disregarded. Upper‐body musculature has higher anaerobic energy production than lower‐body muscles. Indeed, lactate release is higher during arm than leg exercise (Ahlborg and Jensen‐Urstad 1991), and even in highly trained cross‐country skiers, arm oxygen extraction capacity is inferior to leg oxygen extraction capacity (Calbet et al. 2005). Mitochondrial density is lower in *m*. deltoideus than in *m*. vastus lateralis, despite similar fiber type distribution (Kiilerich et al. 2008), which suggests that lactate production and oxidation is higher and lower, respectively, in arm than in leg musculature (Jacobs et al. 2013). It appears likely that the higher reliance on anaerobic metabolism in upper‐body musculature is associated with a higher capacity for acid/base regulation, including a higher expression of the lactate/H^+^ cotransporter monocarboxylate transporter 4 (MCT4) as well as the Na^+^/H^+^ exchanger 1 (NHE1) as compared to lower‐body musculature, but this is yet to be examined.

It is currently unclear how the more glycolytic upper‐body musculature adapts to exercise training with a high anaerobic energy demand. It is well established that high‐intensity interval training (HIT) is a powerful stimulus for increasing the expression of MCT4 and NHE1 in vastus lateralis muscle (Pilegaard et al. 1999; Mohr et al. 2007; Iaia et al. 2008). We have recently demonstrated that the oxidative adaptive response to intense exercise training is higher in *m*. deltoideus than in *m*. vastus lateralis (Nordsborg et al. 2015), which may be related to the initial lower oxidative capacity of arm muscle (Kiilerich et al. 2008; Nordsborg et al. 2015). Thus, it can be speculated that the upper‐body musculature, due to the more anaerobic characteristics, will show less adaptation to high‐intensity training than the more oxidative lower‐body musculature.

Furthermore, it has been observed that resting Na^+^/K^+^ pump activity, determined by use of the K^+^‐stimulated 3‐*O*‐methylfluorescein phosphatase assay (Fraser and McKenna 1998), as well as subunit‐specific mRNA expression, is higher in *m*. vastus lateralis than in *m*. deltoideus, despite similar [^3^H] ouabain binding sites’ determined Na^+^/K^+^ pump concentration (Nordsborg et al. 2005). Thus, it seems plausible that there is a difference in the protein expression of specific Na^+^/K^+^ pump subunits between upper‐ and lower‐body musculature, but this has not been investigated. Moreover, it is well known that Na^+^/K^+^ pump expression increases with HIT in leg muscle (Mohr et al. 2007; Iaia et al. 2008; Bangsbo et al. 2009), but it is not known whether the possible lower subunit‐specific expression in upper‐body musculature and lower training status in a sedentary subject population result in a higher adaptive potential than in locomotor musculature.

It is well known that HIT increases fatigue resistance (Mohr et al. 2007; Iaia et al. 2008; Bangsbo et al. 2009) and that changes in myocyte redox potential may be involved in the development of fatigue during intense exercise (Allen et al. 2008). Thus, improved performance during intense exercise following a period of HIT may be caused by improved handling of reactive oxygen species (ROS). Consequently, it is of interest to investigate whether HIT increases expression of the antioxidative proteins superoxide dismutase 1 (SOD1) and SOD2 as well as catalase (CAT). As high‐intensity exercise training increases SOD2 expression in leg musculature (Gliemann et al. 2013), it seems reasonable to expect that HIT engaging upper‐body muscles also increases expression of antioxidants in arm muscle.

We have previously demonstrated that resting muscle glycogen concentration increased in *m*. deltoideus following a period of high‐intensity interval swimming training, whereas it was unaffected by soccer training in *m*. vastus lateralis (Nordsborg et al. 2015). However, potential underlying mechanisms remain to be elucidated. An increase in *m*. vastus lateralis glycogen content with HIT is accompanied by higher glucose transporter 4 (GLUT4) expression (Little et al. 2010), but it is unclear whether this also occurs in arm musculature. If this is the case, it may partly explain the previously observed increase in glycogen concentration due to a higher uptake of glucose in the recovery period after exercise. In addition, glycogen synthase (GS) activity is known to be a rate‐limiting step in glycogen synthesis (Lawrence and Roach 1997) and to be upregulated with exercise training (Christ‐Roberts et al. 2004). Consequently, the possibility exists that higher GS expression, and thus higher enzymatic capacity to resynthesize glycogen following each HIT session, may have contributed to the previously observed increase in the resting level of glycogen in *m. *deltoideus.

Based on the above considerations, the hypothesis of this study was that upper‐body musculature has a higher capacity for extrusion of H^+^ and lactate than lower‐body musculature and that the adaptation of arm skeletal muscle H^+^ and lactate transporters to exercise training is less than in leg musculature. Additionally, the basal expression of Na^+^/K^+^ pump subunits is hypothesized to be lower in *m*. deltoideus than in *m*. vastus lateralis, thus having a higher adaptive potential to exercise training. Finally, it is hypothesized that markers of antioxidant capacity as well as GLUT4 expression will adapt to high‐intensity training in arm musculature, as previously observed in leg muscle.

## Methods

### Subjects and ethical approval

Eighty‐three sedentary premenopausal women with average (±SD) age, height, weight, and body fat of 45 ± 6 years, 165 ± 6 cm, 80.0 ± 14.1 kg, and 42.6 ± 5.7%, respectively, were recruited for the study. The study procedures were approved by the ethical committee of the Faroe Islands as well as the Sport and Health Sciences Research Ethics Committee at the University of Exeter, Exeter, United Kingdom, and conducted in accordance with the Declaration of Helsinki (1964). After being informed verbally and in writing of the experimental procedures and associated risks, all the participants gave their written consent to take part in the study.

### Experimental design

**Figure 1:**
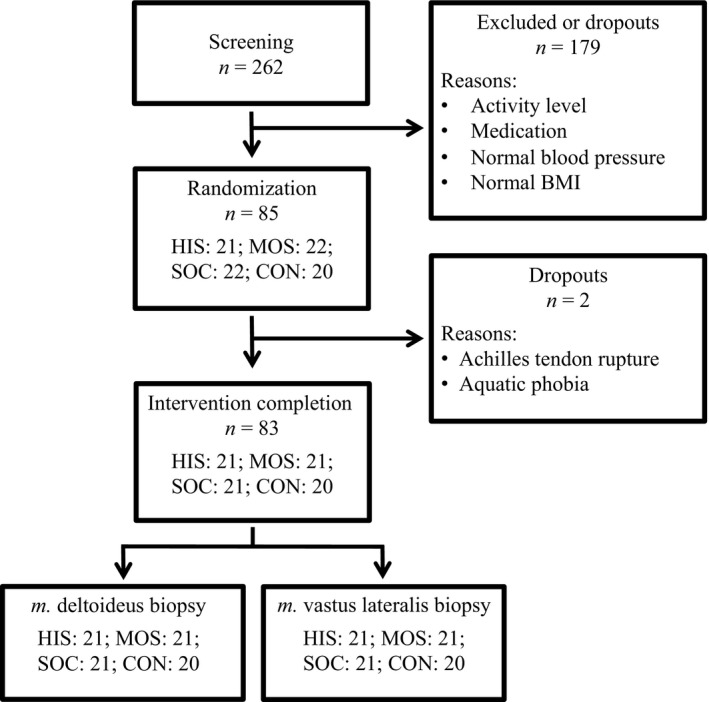
Illustration of the study recruitment process and number of obtained muscle biopsy samples. See text for details. CON, control; SOC, soccer; MOS, moderate‐intensity continuous swimming; HIS, high‐intensity intermittent swimming.

The study was designed as a randomized controlled trial and parts of the obtained data have been reported elsewhere (Mohr et al. 2014a, 2014b; Nordsborg et al. 2015). After initial testing of 262 volunteers, the subjects were enrolled in the study based on selection criteria of a sedentary lifestyle for at least 2 years, mild hypertension (mean arterial pressure of 96–110 mmHg), and body mass index of at least 25 kg/m^2^. The subjects were randomized into one of four intervention groups (Fig. 1): a high‐intensity intermittent swimming group (HIS, *n *=* *21: age 44 ± 5 years; height 164 ± 6 cm; weight 76.5 ± 8.8 kg); a moderate‐intensity continuous swimming group (MOS, *n *=* *21: age 46 ± 4 years; height 165 ± 5 cm; weight 83.8 ± 18.8 kg); a soccer training group (SOC, *n *=* *21: age 45 ± 5 years; height 165 ± 7 cm; weight 79.8 ± 12.8 kg); and a control group (CON, *n *=* *20: age 45 ± 4 years; height 166 ± 6 cm; weight 77.3 ± 10.4 kg). The training groups performed three weekly training sessions (HIS: 2.9 ± 0.5; MOS: 2.9 ± 0.5; and SOC: 3.0 ± 0.4) for 15 weeks, whereas CON had no training or lifestyle changes during the same period. Within 10 days before initiation of the intervention and 48–72 h after the last training session, resting muscle biopsies were obtained from *m*. deltoideus and *m*. vastus lateralis. Subsequently, the muscle tissue samples were analyzed for intervention‐induced changes in protein expression by Western blot analysis. Dietary intake was not controlled during the intervention and the experimental days were not timed in relation to the menstrual cycle.

### Training intervention

HIS completed a total of 44 ± 4 training sessions over the 15‐week intervention period. Each HIS training session lasted ~15–25 min (3–5 min of effective swimming) and consisted of 6–10 × 30‐s all‐out front‐crawl swimming intervals interspersed with 2 min of passive recovery according to training principles previously described (Mohr et al. 2007). In the first 6 weeks of training, each session consisted of six intervals, in the following 6 weeks the sessions consisted of eight intervals, and in the final 3 weeks the number of intervals per session was increased to 10. MOS completed a total of 43 ± 4 training sessions over 15 weeks. All MOS training sessions lasted 1 h and consisted of continuous front‐crawl swimming, with the participants encouraged to swim as far as possible during each session. Five trained swimming coaches were present during all training sessions to control the intensity and duration of the training, ensure a safe training environment, and give technical advice. SOC completed a total of 45 ± 3 training sessions over the 15‐week intervention period. All SOC training sessions lasted 1 h and consisted of small‐sided soccer games (4 vs. 4 to 10 vs. 10) as previously described (Randers et al. 2010). A trained soccer coach was present during all sessions to control the duration of the training and ensure competitive games.

### Muscle sampling

Tissue samples from the medial part of *m*. vastus lateralis and from the posterior (~90% of samples) or anterior (~10% of samples) part of *m. *deltoideus were obtained under local anesthetic (1% lidocaine) using a percutaneous needle biopsy technique (Bergstrom 1962). When palpation of the posterior part of the deltoideus muscle was not possible due to subcutaneous adipose tissue, the muscle biopsy was obtained from the anterior deltoideus, despite possible metabolic differences between the two portions. However, it was assumed that both parts of *m*. deltoideus are active during front‐crawl swimming and have high adaptive potential in sedentary women. Muscle biopsies were obtained from the same portion before and after the intervention. The biopsy tissue samples were immediately frozen in liquid nitrogen and stored at −80°C for subsequent analysis. Due to sampling complications and the analytical process, the number of observations for each time point is lower than the potential 83 (Fig. 1). The number of useful deltoideus muscle biopsy samples was lower, and the samples were generally smaller than samples obtained from vastus lateralis muscle. Changes in protein expression in *m*. deltoideus and in *m*. vastus lateralis were determined from double determinations where possible (~51 and ~81% of samples, respectively), that is, the tissue samples were divided and kept in two parts before freeze‐drying and the mean signal intensity of the two resulting samples was then used as the result for the individual time point. For all results, the number of included observations is provided (see Fig. 1 for participant and analysis details).

### Quantification of protein expression

Muscle preparation, Western blot analysis, and quantification of resulting band signal intensities were performed as previously described (Nordsborg et al. 2015). Briefly, the frozen muscle tissue samples were weighted before and after freeze‐drying to determine the water content. Freeze‐dried samples of ~2.5 mg dry weight were carefully dissected free from visible blood, fat, and connective tissue and homogenized in lysis buffer. After centrifugation, the protein concentration of the supernatant (lysate) was determined using a bovine serum albumin (BSA) standard kit (Pierce, Rockford, IL) and samples were diluted with 6× Laemmli buffer and ddH_2_O to achieve equal protein concentration. Equal amounts of total protein were loaded in each well of precast gels (Bio‐Rad Laboratories). A standard curve representative of all the study samples was loaded on two gels to verify that the resulting band signal intensities were located on the antibody‐specific standard curve. All samples from one subject were loaded side by side on the same gel to avoid any potential bias as a result of variation in transfer efficiency across and between gels. Likewise, for most of the gels used all intervention groups were represented evenly and randomly placed across each gel. Lysate proteins were separated according to molecular weight by sodium dodecyl sulfate–polyacrylamide gel electrophoresis (SDS‐PAGE) and transferred semidry to a polyvinylidene difluoride (PVDF) membrane (Bio‐Rad). The membranes were blocked in either 3% BSA or 2% nonfat milk in Tris‐buffered saline including 0.1% Tween‐20 (TBST) before overnight incubation in primary antibody at 4°C and subsequent 1‐h incubation in secondary antibody at room temperature. The membrane staining was visualized by incubation with a chemiluminescent horseradish peroxide (HRP) substrate (ECL, Millipore) before image digitalization (ChemiDoc MP Imaging System, Bio‐Rad Laboratories). Image Lab v. 4.0 (Bio‐Rad Laboratories) was used for densitometry quantification of the Western blot band signal intensity and adjustment for background intensity. Individual after versus before as well as *m*. vastus lateralis versus *m*. deltoideus ratios for band signal intensity were calculated, and resulting values ≥ 3 were excluded to avoid type II error. It should be noted that no deglycosylation procedure was performed as pilot studies revealed that the expression analysis was unaffected by the shift in molecular weight induced by a deglycosylation step (data not shown).

### Antibodies

**Figure 2:**
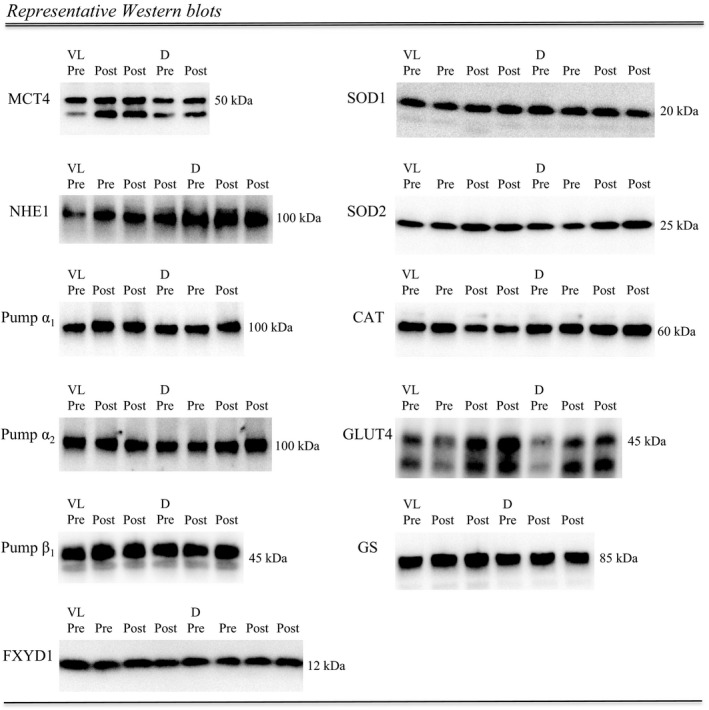
Representative Western blots for a subject in the high‐intensity intermittent swimming training group, including the molecular weight of band migration. Muscle tissue samples were obtained before (Pre) and after (Post) the intervention period and the protein expression was determined in duplicate when possible (see “muscle sampling” in the methods section for details). VL, vastus lateralis muscle; D, deltoideus muscle; MCT4, monocarboxylate transporter 4; NHE1, Na+/H+ exchanger 1. Pump *α*1, *α*2, and *β*1 =  Na+/K+ pump *α*1, *α*2, and *β*1 subunit, respectively. FXYD1, phospholemman; SOD1 and SOD2, superoxide dismutase 1 and 2, respectively. CAT, catalase; GLUT4, glucose transporter 4; GS, glycogen synthase.

The primary antibodies were optimized by use of a serial dilution of mixed human muscle standard lysate to ensure that the band signal intensities were located on the linear part of the antibody‐specific standard curve. Primary antibodies targeting the investigated Na^+^/K^+^ pump subunits and phospholemman (FXYD1) were monoclonal Na^+^/K^+^ pump *α*_1_ (alfa6F, Developmental Study Hybridoma Bank, University of Iowa, United states of America), polyclonal *α*_2_ (07‐674, Millipore), monoclonal *β*_1_ (MA3‐930, Thermo Scientific), and polyclonal FXYD1 (13721‐1‐AP, Datasheet). Polyclonal MCT4 (AB3316P, Millipore) and monoclonal NHE1 (MAB3140, Chemicon) were used to detect the expression of MCT4 and NHE1. Applied antibodies targeting antioxidative and glycogen‐level‐related proteins were polyclonal SOD1 (574597, Millipore) and polyclonal SOD2 (06‐984, Millipore) (Kindly provided by Prof. H. Pilegaard, University of Copenhagen), polyclonal KAT (ab1877, Abcam), polyclonal GLUT4 (PA1‐1065, Thermo Fisher Scientific), and polyclonal GS (3893, Cell Signaling Technology). The secondary antibodies used were HRP‐conjugated goat anti‐rabbit (4010‐05, Southern Biotech), rabbit anti‐sheep (P‐0163, DAKO), and goat anti‐mouse (P‐0447, DAKO). A representative example of Western blotting is provided in Fig. 2.

### Data treatment and statistical analysis

Data are presented as means ± SD. Specific statistical analyses (using SPSS v. 23, IBM) were applied to answer the primary hypotheses. To test for possible changes in protein expression within a specific muscle and intervention group, individual after versus before intervention ratios were calculated and a one‐sample *t*‐test was used to evaluate whether the investigated group and muscle mean was ≠ 1. A mixed‐model approach using the SPSS MIXED procedure (Cnaan et al. 1997) was used to test for possible differences in protein expression changes within muscle between groups and within group between muscles. Muscle and group were specified as fixed effects, whereas random variation and repeated effects were specified by subject. When a significant main effect or interaction was detected, a multiple‐comparisons approach with Sidak adjustment was applied. To specifically address whether the pre‐ compared to postintervention ratios in vastus lateralis muscle after SOC and in deltoideus muscle after MOS and HIS were different, group and muscle were combined into a single variable and a mixed model with “GroupMuscle” as a fixed factor and subject as a random factor and repeated effect, was applied. When a significant main effect was observed, a multiple‐comparisons approach with no adjustment (to avoid type II error) was used to identify the difference(s). The level of significance was set at *P *<* *0.05, and *P *<* *0.1 is reported as a tendency.

## Results

### Muscle ion transport proteins

There were marked differences in expression of the investigated ion transporters between arm and leg muscles before initiation of the training intervention. Specifically, the expression of MCT4 was 41 ± 69% higher (*P *<* *0.001; *n *=* *46) in vastus lateralis muscle than in deltoideus muscle, while the expression of NHE1 was 30 ± 51% lower in *m*. vastus lateralis (*P *<* *0.001; *n *=* *51). Na^+^/K^+^ pump *α*_2_ expression was 23 ± 63% higher (*P *<* *0.01; *n *=* *54) in vastus lateralis muscle than in deltoideus muscle, whereas no difference was observed between the two muscles’ *α*_1_ subunit (−1 ± 63%; *P *=* *0.90; *n *=* *51), *β*_1_ subunit (2 ± 33%; *P *=* *0.61; *n *=* *58), or FXYD1 (6 ± 67%; *P *=* *0.54; *n *=* *41) expression.

**Figure 3:**
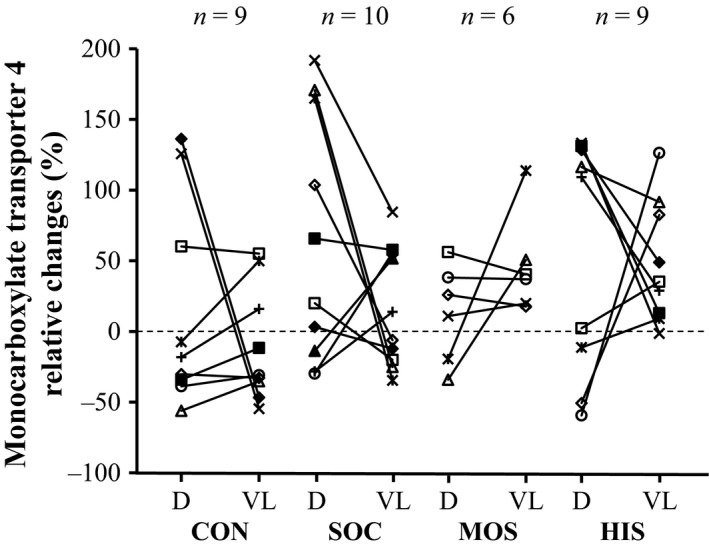
Individual changes (post relative to pre) in protein expression of the monocarboxylate transporter 4 determined in muscle tissue obtained from the arms (deltoideus muscle, D) and the legs (vastus lateralis muscle, VL) before and after an intervention period consisting of control (CON), soccer (SOC), moderate‐intensity continuous swimming (MOS), and high‐intensity intermittent swimming (HIS).

As a result of the training intervention, the expression of all ion transport proteins was elevated in either one or both muscle groups (Table 1). Specifically, deltoid MCT4 expression was increased in HIS (*P *<* *0.05) and SOC (*P *<* *0.05) but unaltered in MOS and CON (Fig. 3). In addition, when MOS and HIS were pooled, a 42 ± 72% increase (*P *<* *0.05; *n *=* *20) was observed. Vastus lateralis muscle MCT4 expression was upregulated after HIS (*P *<* *0.01), MOS (*P *<* *0.01) and SOC (*P *<* *0.05), while no change was apparent in CON.

**Table 1:** Protein expression (post relative to pre intervention expression)

	Intervention group			
	CON	SOC	MOS	HIS
Monocarboxylate transporter 4				
D	18 ± 67% (*n *=* *11)	62 ± 88% (*n *=* *12)*	9 ± 40% (*n *=* *8)	63 ± 82% (*n *=* *12)*
VL	9 ± 53% (*n *=* *14)	38 ± 63% (*n *=* *16)*	47 ± 47% (*n *=* *17)**	51 ± 60% (*n *=* *16)**
D versus VL	ns (*n *=* *9)	ns (*n *=* *10)	ns (*n *=* *6)	ns (*n *=* *9)
versus CON		ns	ns	ns
Na^+^/H^+^ exchanger 1				
D	−11 ± 56% (*n *=* *14)	9 ± 68% (*n *=* *13)	8 ± 76% (*n *=* *11)	5 ± 70% (*n *=* *15)
VL	8 ± 27% (*n *=* *13)	25 ± 77% (*n *=* *14)	12 ± 38% (*n *=* *19)	55 ± 74% (*n *=* *15)*
D versus VL	ns (*n *=* *10)	ns (*n *=* *8)	ns (*n *=* *11)	ns (*n *=* *10)
versus CON		ns	ns	ns
Na^+^/K^+^ pump *α*_1_				
D	50 ± 76% (*n *=* *13)*	41 ± 74% (*n *=* *10)	19 ± 49% (*n *=* *11)	21 ± 63% (*n *=* *12)
VL	−1 ± 49% (*n *=* *15)	65 ± 55% (*n *=* *15)***	25 ± 52% (*n *=* *16; *P *=* *0.07)	32 ± 65% (*n *=* *14; *P *=* *0.08)
D versus VL	§§ (*n *=* *11)	ns (*n *=* *8)	ns (*n *=* *10)	ns (*n *=* *8)
versus CON		VL:$$	ns	ns
Na^+^/K^+^ pump *α*_2_				
D	20 ± 42% (*n *=* *13)	20 ± 66% (*n *=* *11)	24 ± 51% (*n *=* *13)	30 ± 51% (*n *=* *15)*
VL	−5 ± 36% (*n *=* *15)	−8 ± 28% (*n *=* *15)	9 ± 45% (*n *=* *20)	20 ± 60% (*n *=* *18)
D versus VL	ns (*n *=* *11)	ns (*n *=* *9)	ns (*n *=* *13)	ns (*n *=* *12)
versus CON		ns	ns	ns
Na^+^/K^+^ pump *β*_1_				
D	7 ± 35% (*n *=* *15)	2 ± 28% (*n *=* *14)	5 ± 26% (*n *=* *13)	−1 ± 17% (*n *=* *16)
VL	6 ± 32% (*n *=* *15)	13 ± 37% (*n *=* *17)	15 ± 22% (*n *=* *20)**	12 ± 29% (*n *=* *18; *P *=* *0.09)
D versus VL	ns (*n *=* *13)	ns (*n *=* *12)	ns (*n *=* *13)	ns (*n *=* *13)
versus CON		ns	ns	ns
FXYD1				
D	51 ± 94% (*n *=* *9)	5 ± 53% (*n *=* *11)	6 ± 78% (*n *=* *10)	39 ± 81% (*n *=* *12)
VL	15 ± 69% (*n *=* *12)	16 ± 45% (*n *=* *14)	42 ± 56% (*n *=* *14)*	9 ± 48% (*n *=* *15)
D versus VL	ns (*n *=* *7)	ns (*n *=* *7)	ns (*n *=* *7)	ns (*n *=* *9)
versus CON		ns	ns	ns
Superoxide dismutase 1				
D	11 ± 42% (*n *=* *15)	−4 ± 38% (*n *=* *13)	−3 ± 44% (*n *=* *14)	−16 ± 44% (*n *=* *16)
VL	14 ± 45% (*n *=* *15)	1 ± 39% (*n *=* *17)	11 ± 43% (*n *=* *20)	36 ± 48% (*n *=* *17)**
D versus VL	ns (*n *=* *13)	ns (*n *=* *11)	ns (*n *=* *14)	§ (*n *=* *12)
versus CON		ns	ns	ns
Superoxide dismutase 2				
D	15 ± 39% (*n *=* *15)	32 ± 57% (*n *=* *13; *P *=* *0.06)	29 ± 56% (*n *=* *12)	66 ± 70% (*n *=* *16)**
VL	19 ± 39% (*n *=* *15; *P *=* *0.08)	44 ± 39% (*n *=* *17)***	57 ± 32% (*n *=* *20)***	63 ± 54% (*n *=* *17)***
D versus VL	ns (*n *=* *13)	ns (*n *=* *11)	ns (*n *=* *12)	ns (*n *=* *13)
versus CON		ns	ns	VL:$$; D: *P *=* *0.08
Catalase				
D	−24 ± 56% (*n *=* *12)	6 ± 77% (*n *=* *11)	2 ± 71% (*n *=* *11)	4 ± 76% (*n *=* *14)
VL	8 ± 47% (*n *=* *13)	12 ± 85% (*n *=* *15)	−12 ± 40% (*n *=* *20)	25 ± 79% (*n *=* *13)
D versus VL	ns (*n *=* *9)	ns (*n *=* *9)	ns (*n *=* *11)	ns (*n *=* *9)
versus CON		ns	ns	ns
Glucose transporter 4 (GLUT4)				
D	24 ± 52% (*n *=* *14)	52 ± 60% (*n *=* *13)**	50 ± 72% (*n *=* *11)*	55 ± 58% (*n *=* *15)**
VL	−4 ± 29% (*n *=* *15)	43 ± 53% (*n *=* *17)**	40 ± 37% (*n *=* *19)***	38 ± 49% (*n *=* *17)**
D versus VL	ns (*n *=* *12)	ns (*n *=* *11)	ns (*n *=* *10)	ns (*n *=* *11)
versus CON		VL: *P *=* *0.07	ns	ns
Glycogen synthase				
D	41 ± 61% (*n *=* *12)*	48 ± 81% (*n *=* *12; *P *=* *0.07)	38 ± 60% (*n *=* *11; *P *=* *0.06)	22 ± 55% (*n *=* *15)
VL	20 ± 57% (*n *=* *15)	5 ± 35% (*n *=* *16)	21 ± 73% (*n *=* *19)	35 ± 59% (*n *=* *17)*
D versus VL	ns (*n *=* *10)	ns (*n *=* *9)	ns (*n *=* *11)	ns (*n *=* *12)
versus CON		ns	ns	ns

Results are means ± SD. D, deltoideus muscle; VL, vastus lateralis muscle; CON, no physical activity; SOC, soccer; MOS, moderate‐intensity continuous swimming; HIS, high‐intensity intermittent swimming; ns, nonsignificant. Significant change within a muscle group and intervention group: *P <* *0.05, **P <* *0.01, and ***P <* *0.001. Significant difference between changes in muscle groups within an intervention group: ^§^ *P *<* *0.05 and ^§§^ *P *<* *0.01. Significant difference compared to the control group within the same muscle group: ^$$^ *P *<* *0.01.

With respect to NHE1, none of the applied training regimes altered the expression of NHE1 in *m*. deltoideus. Likewise, when the two swimming groups were combined, deltoid NHE1 expression remained unchanged (6 ± 71%; *P *=* *0.65; *n *=* *26). In contrast, vastus lateralis muscle NHE1 expression was increased (*P *<* *0.05) in HIS but unaltered in SOC, MOS, and CON.

In deltoideus muscle, Na^+^/K^+^ pump *α*_1_ expression was increased (*P *<* *0.05) in CON but remained similar to the pre‐intervention level in the training groups. Furthermore, the pre compared to post ratios in *m*. deltoideus *α*_1_ isoform expression in SOC, MOS, and HIS did not differ from the change in CON. Notably, when analyzing the response of the combined swimming groups, the expression of Na^+^/K^+^ pump *α*_1_ in *m*. deltoideus tended (*P *=* *0.09; *n *=* *23) to be elevated by 20 ± 56% as a result of training. Vastus lateralis muscle Na^+^/K^+^ pump *α*_1_ expression was increased (*P *<* *0.001) in SOC and tended to increase in MOS (*P *=* *0.07) and in HIS (*P *=* *0.08), while no change was observed in CON. Furthermore, the training‐induced change in *m*. vastus lateralis *α*_1_ isoform expression after SOC was larger (*P *<* *0.01) than the similar protein expression observed before and after the intervention in CON (Table 1).

**Figure 4:**
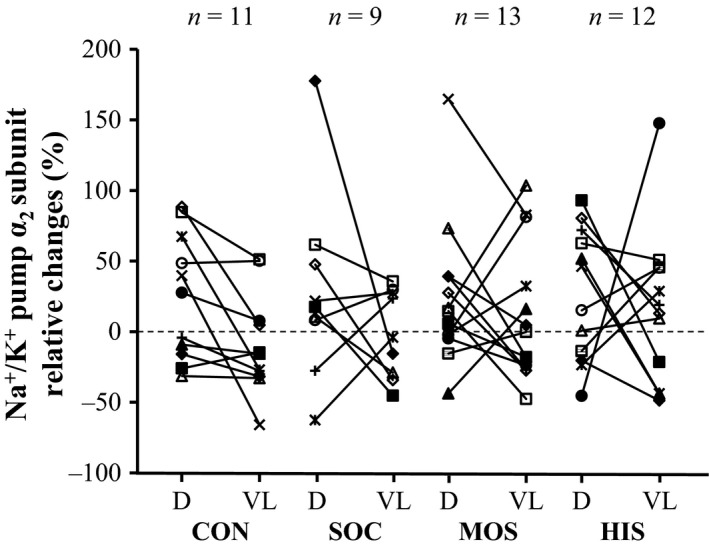
Individual changes (post relative to pre) in protein expression of the Na^+^/K^+^ pump *α*_2_ subunit determined in muscle tissue obtained from the arms (deltoideus muscle, D) and the legs (vastus lateralis muscle, VL) before and after an intervention period consisting of control (CON), soccer (SOC), moderate‐intensity continuous swimming (MOS), and high‐intensity intermittent swimming (HIS).

Deltoideus muscle Na^+^/K^+^ pump *α*_2_ expression was increased (*P *<* *0.05) in HIS but unchanged in the other intervention groups and in CON (Fig. 4). Notably, a 27 ± 50% increase (*P *<* *0.01; *n *=* *28) in *α*_2_ subunit expression was observed when including both swimming groups in the analysis. Vastus lateralis muscle Na^+^/K^+^ pump *α*_2_ expression remained similar to the pre‐intervention level in the training groups and in CON. Additionally, the training‐induced increase in deltoideus muscle *α*_2_ subunit expression after HIS differed (*P *<* *0.05) from the similar level observed in *m*. vastus lateralis before and after SOC. Moreover, the pre‐ to postintervention ratio in *m*. deltoideus *α*_2_ isoform expression after MOS tended (*P *=* *0.06) to be larger than in *m*. vastus lateralis after SOC. It may be noted that there was no difference in the pre‐ to post‐training ratio of *m*. deltoideus Na^+^/K^+^ pump *α*_2_ expression between MOS and HIS.

In comparison with the pre‐intervention level, deltoideus muscle Na^+^/K^+^ pump *β*_1_ expression was similar after training in all intervention groups and in CON. Notably, when the swimming groups were pooled, *m*. deltoideus *β*_1_ subunit expression was not affected by training (2 ± 21%; *P *=* *0.65; *n *=* *29). In *m*. vastus lateralis, *β*_1_ isoform expression was increased (*P *<* *0.01) in MOS, tended (*P *=* *0.09) to increase in HIS, and was unaltered in SOC as well as in CON.

The expression of FXYD1 in *m*. deltoideus was unchanged in the training groups and in CON. Furthermore, when the HIS and MOS responses were combined, deltoid FXYD1 expression remained unaffected by training (24 ± 80%; *P *=* *0.17; *n *=* *22). In vastus lateralis muscle, the expression of FXYD1 was increased (*P *<* *0.05) in MOS but was similar to the pre‐intervention level after SOC, HIS, and CON.

Additionally, no within‐group differences were observed between *m*. deltoideus and *m*. vastus lateralis pre‐ to postintervention ratios for the expression of ion transporters (Table 1). Moreover, when including both swimming groups in the analysis, there were no differences in pre to post ratios between vastus lateralis muscle and deltoid expression for MCT4 (48 ± 39 vs. 38 ± 70%; *P *=* *0.71; *n *=* *15), NHE1 (19 ± 60 vs. 4 ± 71%; *P *=* *0.42; *n *=* *21), Na^+^/K^+^ pump *α*_1_ (22 ± 54 vs. 25 ± 60%; *P *=* *0.90; *n *=* *18), Na^+^/K^+^ pump *α*_2_ (14 ± 51 vs. 25 ± 48%; *P *=* *0.46; *n *=* *25), or FXYD1 (40 ± 54 vs. 24 ± 83%; *P *=* *0.54; *n *=* *16), while the pre to post ratio of *m*. vastus lateralis Na^+^/K^+^ pump *β*_1_ expression only tended (*P *=* *0.097; *n *=* *26) to be larger than in *m*. deltoideus (14 ± 23 vs. 1 ± 22%). Finally, no differences between pre‐ to post‐training ratios in vastus lateralis muscle after SOC and in deltoideus muscle after MOS and HIS were detectable for the expression of MCT4, NHE1, Na^+^/K^+^ pump *α*_1,_ Na^+^/K^+^ pump *β*_1_, or FXYD1.

### Muscle antioxidative proteins

Of the investigated antioxidants, only SOD2 expression differed between arm and leg muscles before the training period. Specifically, the expression of SOD2 was 19 ± 55% higher (*P *<* *0.05; *n *=* *58) in vastus lateralis muscle than in deltoideus muscle, whereas no difference was observed between muscle groups for the expression of either SOD1 (−8 ± 43%; *P *=* *0.14; *n *=* *57) or CAT (−12 ± 60%; *P *=* *0.16; *n *=* *50).

**Figure 5:**
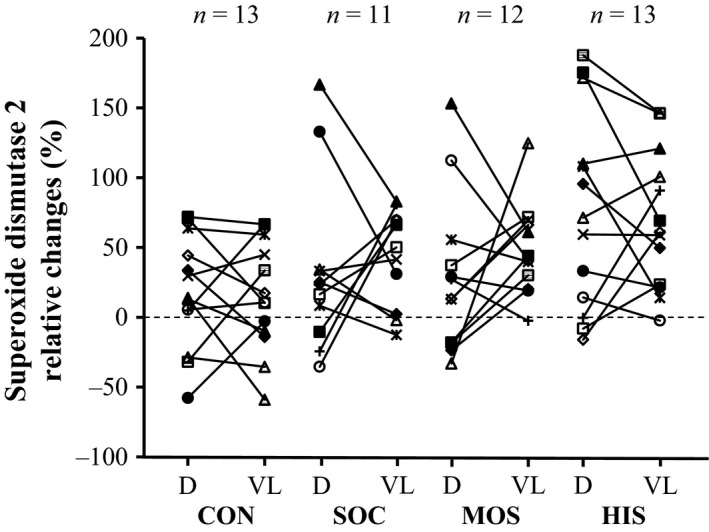
Individual changes (post relative to pre) in protein expression of the superoxide dismutase 2 determined in muscle tissue obtained from the arms (deltoideus muscle, D) and the legs (vastus lateralis muscle, VL) before and after an intervention period consisting of control (CON), soccer (SOC), moderate‐intensity continuous swimming (MOS), and high‐intensity intermittent swimming (HIS).

The expression of SOD1 and SOD2 was increased by training in either one or both muscle groups, whereas the expression of CAT was unaffected by the intervention (Table 1). Specifically, deltoideus muscle SOD2 expression was increased (*P *<* *0.01) in HIS, tended (*P *=* *0.06) to increase in SOC, and was unchanged in MOS as well as in CON (Fig. 5). Notably, a 50 ± 66% increase (*P *<* *0.001; *n *=* *28) in deltoideus muscle SOD2 expression was observed when including both swimming groups in the analysis. Furthermore, the intervention‐induced increase in SOD2 expression after HIS tended (*P *=* *0.08) to be higher than in CON. With respect to *m. *vastus lateralis, SOD2 expression was increased in SOC (*P *<* *0.001), HIS (*P *<* *0.001), and MOS (*P *<* *0.001) and tended (*P *=* *0.08) to increase in CON. Moreover, the change in HIS was larger (*P *<* *0.01) than in CON (Table 1).

Deltoideus muscle SOD1 and CAT expression remained similar to the level observed before initiation of the intervention in both the training groups and in CON. In addition, when analyzing the response of the combined swimming groups, deltoid SOD1 and CAT expression remained unaffected by training (−10 ± 44%; *P *=* *0.22; *n *=* *30 and 3 ± 73%; *P *=* *0.84; *n *=* *25, respectively). In *m*. vastus lateralis, the expression of CAT was unresponsive to the applied training protocols. On the other hand, an increase (*P *<* *0.01) in vastus lateralis muscle SOD1 expression was observed in HIS, while no changes were apparent in SOC, MOS, and CON. Within the HIS group, the training‐induced change in vastus lateralis muscle SOD1 expression was higher (*P *<* *0.05; *n *=* *12) than the pre‐ to post‐training ratio observed in deltoid (32 ± 43 vs. −8 ± 48%) (Table 1). Notably, when HIS and MOS were pooled, the pre‐ to postintervention ratio of *m*. vastus lateralis SOD1 expression was larger (*P *<* *0.05; *n *=* *26) than that of *m*. deltoideus (23 ± 46 vs. −5 ± 45%), whereas the pre to post ratios of SOD2 and CAT expression did not differ between deltoid and vastus lateralis muscle (59 ± 43 vs. 54 ± 68%; *P *=* *0.67; *n *=* *25 and 4 ± 67 vs. 2 ± 75%; *P *=* *0.93; *n *=* *20, respectively). Finally, with respect to the expression of the antioxidants, no differences between pre compared to post ratios in deltoideus muscle after MOS and HIS and in vastus lateralis muscle after SOC were detectable.

### Resting glycogen‐level‐related proteins

Basal GLUT4 expression was 49 ± 52% higher (*P *<* *0.001; *n *=* *52) in vastus lateralis muscle than in deltoideus muscle, whereas the expression of GS was similar (14 ± 60%; *P *=* *0.102; *n *=* *53).

In *m*. deltoideus and in *m*. vastus lateralis, the expression of GLUT4 was increased in SOC (*P *<* *0.01), MOS (*P *<* *0.05; *P *<* *0.001, respectively), and HIS (*P *<* *0.01) but unchanged in CON (Table 1). Notably, when the HIS and MOS groups were combined, a 53 ± 63% increase (*P *<* *0.001; *n *=* *26) in deltoideus muscle GLUT4 expression was observed. Moreover, in vastus lateralis muscle, the change in SOC tended (*P *=* *0.07) to be larger than in CON.

Deltoideus muscle GS expression was increased (*P *<* *0.05) in CON, tended to increase in MOS (*P *=* *0.06) and SOC (*P *=* *0.07), but was unaltered in HIS. However, when both swimming groups were included in the analysis, a 29 ± 57% increase (*P *<* *0.05; *n *=* *26) was detectable. The similar levels of deltoid GS expression observed before and after SOC, MOS, and HIS were not significantly different from the change in CON. In vastus lateralis muscle, an increase (*P *<* *0.05) in the expression of GS was detected in HIS, while no change was apparent in SOC, MOS, and CON.

Additionally, no within‐group differences in pre‐ to postintervention ratios between *m*. deltoideus and *m*. vastus lateralis GLUT4 or GS expression were observed (Table 1). Likewise, when HIS and MOS were combined and the response examined, there were no differences in pre to post ratios of GLUT4 or GS expression between vastus lateralis muscle and deltoideus muscle (32 ± 40 vs. 43 ± 59%; *P *=* *0.52; *n *=* *21 and 31 ± 69 vs. 31 ± 56%; *P *=* *0.99; *n *=* *23, respectively). Finally, no differences were detectable between pre to post ratios of GLUT4 or GS expression in deltoideus muscle after MOS and HIS and in vastus lateralis muscle after SOC.

## Discussion

The primary findings of the present study were that basal expression of MCT4, Na^+^/K^+^ pump *α*_2_, SOD2, and GLUT4 were lower in arm than in leg musculature, whereas the expression of NHE1 was higher. Moreover, it was demonstrated that upper‐body musculature, compared to lower‐body musculature, has a higher potential for increasing Na^+^/K^+^ pump *α*_2_ expression. In contrast, lower‐body musculature showed increasing expression of Na^+^/K^+^ pump *β*_1_, FXYD1, NHE1, and SOD1, which did not occur in the deltoid. However, the apparent difference in response between the muscle groups did not reach statistical significance except for SOD1, where the change in vastus lateralis muscle after HIS was higher than the pre to post ratio in the deltoid muscle. Additionally, high‐intensity interval training, as opposed to moderate‐intensity continuous training, resulted in increased expression of MCT4, Na^+^/K^+^ pump *α*_2_, and SOD2 in arm muscle.

### Basal level of ion transporters and antioxidants in arm versus leg muscle

The present study is the first to compare the expression of proteins related to capacity for handling ROS, La^−^, H^+^, Na^+^, and K^+^ between upper‐ and lower‐extremity muscle groups. Contrary to our hypothesis, basal MCT4 expression was lower in *m*. deltoideus than in *m*. vastus lateralis, which suggests that upper‐body musculature is associated with a lower capacity for lactate handling compared to human locomotor musculature, despite higher exercise carbohydrate oxidation and lower oxidative capacity (Ahlborg and Jensen‐Urstad 1991; Kiilerich et al. 2008; Nordsborg et al. 2015). Moreover, it appears likely that higher exercise lactate release from arm than leg muscle groups (Ahlborg and Jensen‐Urstad 1991) is not due to a larger capacity for transsarcolemmal lactate transport in arm musculature. On the other hand, basal NHE1 expression was larger in deltoid than in *m*. vastus lateralis, and hence, the capacity for Na^+^‐dependent H^+^ extrusion seems larger in upper‐body muscle groups.

The expression of Na^+^/K^+^ pump isoforms was similar between arm and leg muscles except for *α*_2_ subunits, where the expression was lower in deltoideus muscle than in vastus lateralis muscle. It appears that lower Na^+^/K^+^ pump *α*_1_ and *β*_1_ mRNA expression in arm than in leg muscle (Nordsborg et al. 2005) does not result in lower subunit protein expression. Moreover, it can be speculated that lower expression of the *α*_2_ isoform may partly explain lower resting Na^+^/K^+^ pump activity in upper‐body than in lower‐body musculature despite similar pump content (Nordsborg et al. 2005).

The expression of antioxidants did not differ between arm and leg muscles except for SOD2, which was lower in *m*. deltoideus. Thus, it seems that arm musculature exhibits a lower initial capacity for ROS handling. Exercise‐induced oxidative stress originates from mitochondrial respiration (Powers et al. 2011). As the SOD2 protein is located in the mitochondrial matrix (Powers et al. 2011) and lower protein expression of SOD2 in arm than in leg musculature, and consequently lower antioxidant capacity, may thus be a result of lower mitochondrial density (Kiilerich et al. 2008). This suggestion is consistent with the observation of higher PGC‐1alpha, SIRT1, and SIRT3 protein expression in leg than in arm musculature (Ponce‐González et al. 2016) as well as higher expression of citrate synthase and 3‐hydroxyacyl‐CoA dehydrogenase in leg than in arm musculature (Ara et al. 2011).

All in all, the observation that basal MCT4, Na^+^/K^+^ pump *α*_2_, and SOD2 expression are lower in arm than in leg muscle may be due to lower daily contractile activity with upper‐ than lower‐body extremities. However, our finding of larger basal expression of NHE1 in arm than in leg muscle indicates that the difference in protein expression between upper‐ and lower‐body muscle groups is not solely a result of the difference in training status. Instead, higher capacity for Na^+^‐dependent H^+^ extrusion in arm muscle may be a consequence of higher reliance on anaerobic metabolism in arm muscle. Notably, maximal citrate synthase activity in *m*. deltoideus is lower, compared to in *m*. vastus lateralis, in trained athletes (Flynn et al. 1987), suggesting that maximal attainable oxidative capacity is lower in arm muscle.

### Adaptability of arm versus leg musculature

High‐intensity interval swimming and small‐sided soccer games are expected to require a high level of anaerobic energy production (Mohr et al. 2007; Randers et al. 2010). Soccer practice was chosen to last 1 h to ensure that a possible higher adaptive potential of *m*. deltoideus was not due to insufficient training stimulus of *m. *vastus lateralis. The finding of similar increases in MCT4, SOD2, and GLUT4 expression suggests that the adaptability of arm and leg muscles is comparable. However, the observation of training‐induced increases occurring exclusively in leg muscle or arm muscle indicates that the adaptive response differs between upper‐ and lower‐body extremity musculature. Notably, deltoideus muscle GS and Na^+^/K^+^ *α*_1_ expressions were unaltered in the training groups, but not when HIS and MOS were pooled, in which case GS expression was upregulated by 29% and *α*_1_ subunit expression tended (*P *=* *0.09) to be 20% larger after than before training. The increases in MCT4 and SOD2 expression in arm muscle presented in the current study are larger in comparison with findings from leg muscle after a period of intense training (Pilegaard et al. 1999; Gliemann et al. 2013), whereas the increases in GLUT4 and *β*_1_ and *α*_2_ subunit expression are smaller (Gibala et al. 2006; Mohr et al. 2007). Interestingly, FXYD1 and NHE1 expression were unaltered by the applied intense training regimes, contrary to previous findings (Mohr et al. 2007; Thomassen et al. 2016), and SOD1 expression appears to be unaffected by intense training (Gliemann et al. 2013). Thus, the present results demonstrate that there are marked differences in adaptability between arm and leg muscles for all investigated proteins except MCT4, SOD2, and GLUT4 as well as CAT, which was unresponsive to training. Thus, caution should be exercised when extrapolating results from lower‐body to upper‐body muscle groups. Notably, there are also different adaptation patterns in oxidative capacity between *m*. deltoideus and *m*. vastus lateralis (Helge et al. 2008; Nordsborg et al. 2015).

Lower basal oxidative capacity coincides with a higher adaptive potential (Helge et al. 2008; Nordsborg et al. 2015). In contrast, lower MCT4, SOD2, and GLUT4 expression in arm than in leg muscle before the intervention did not result in higher adaptability of upper‐body muscle groups. On the other hand, initial NHE1 expression was larger in arm muscle and was upregulated solely in vastus lateralis muscle, while basal Na^+^/K^+^ pump *α*_2_ expression was lower in arm muscle and affected only in deltoideus muscle by high‐intensity interval swimming. In addition, there was no difference between arm and leg muscle Na^+^/K^+^ pump *α*_1_ and *β*_1_, FXYD1, GS, and SOD1 expression before initiation of the intervention, yet the level of these proteins was upregulated in *m*. vastus lateralis but not in *m*. deltoideus as a result of training.

Taken together, the difference in adaptive responses to exercise training between upper‐body and lower‐body muscle groups seems to be unrelated to the initial expression except for NHE1 and Na^+^/K^+^ pump *α*_2_, where it is possible that lower basal expression may have caused higher adaptability. It may be speculated that the potential for increasing MCT4, SOD2, and GLUT4 expression was higher in arm than in leg muscle, as the total time spent training was ~10 times higher for the SOC group than for the HIS group. However, as the contraction pattern between *m*. deltoideus and *m*. vastus lateralis most likely differs, our setup does not allow this conclusion to be drawn.

With respect to our recent finding of increased glycogen availability in *m*. deltoideus after HIS (Nordsborg et al. 2015), the present results demonstrate that GLUT4 expression was upregulated to a similar extent in deltoid after HIS and in *m. *vastus lateralis after SOC. In addition, deltoideus muscle GS expression was unaltered in HIS; hence, it appears that dynamics other than increased postexercise glucose uptake and glycogen synthesis capacity are associated with the training‐induced increase in glycogen content in arm muscle.

### Influence of exercise training intensity on adaptations in arm muscle

The training volume differed markedly between MOS and HIS. The MOS group spent 1‐h swimming per training session, while the HIS group completed a total of 5 min of intense exercise per session. Consequently, comparison of the adaptive response in deltoideus muscle between HIS and MOS makes it possible to determine the effect of high or moderate exercise training intensity on adaptations in arm muscle. To the authors’ knowledge, this is the first study to investigate whether moderate‐ and high‐intensity training alters the level of ion transporters and antioxidative proteins in arm muscle. Given the study participant's sedentary lifestyle, it was expected that the potential for adaptations in arm muscle was high. If moderate‐intensity high‐volume training has the potential to increase expression of the investigated proteins, it was therefore anticipated that it would occur with the present setup. However, the expression of Na^+^/K^+^ pump *α*_2_, MCT4, and SOD2 was elevated only after HIS, despite that basal protein expression of all three proteins being lower in arm than in leg muscle and thus having high adaptive potential. It should be noted that GLUT4 expression was upregulated in both swimming groups.

Overall, the observation that only intense exercise training resulted in increased capacity for ion and ROS handling may lead to speculation that significant exercise‐induced fluctuations in ion homeostasis (Mohr et al. 2007) and redox potential (Bailey et al. 2007) are necessary for increases in expression of antioxidants and ion transport proteins to occur as a result of training. However, the finding of elevated MCT4 expression in *m*. deltoideus after SOC as well as unaltered phosphofructokinase maximal activity after intense training (Nordsborg et al. 2015) suggests that muscular adaptations are not necessarily related to the prevailing exercise metabolism.

### Adaptations in nonprimarily engaged muscle groups

During freestyle swimming, *m*. deltoideus is strongly activated, while *m*. vastus lateralis activation is moderate, as evaluated by electromyography measurements of *m*. rectus femoris (Ikuta et al. 2012). On the other hand, vastus lateralis muscle activation is high in small‐sided soccer games, as glycogen depletion occurs in all fiber types (Randers et al. 2010), while deltoideus muscle contracts against external load only during between‐player interactions, which is limited (Mohr et al. 2003). Moreover, exercise training with one leg increases succinate dehydrogenase maximal activity in the trained but not in the contralateral leg (Saltin et al. 1976; Henriksson 1977). With these considerations in mind, it was expected that adaptations would occur only in the investigated prime mover muscle groups. However, expression of MCT4 and GLUT4 was increased in deltoid after SOC and the level of all investigated proteins except CAT and *α*_2_ and *α*_1_ subunits was upregulated in vastus lateralis muscle after HIS and/or MOS. Notably, we recently demonstrated that oxidative capacity was increased in *m*. deltoideus after soccer and in *m*. vastus lateralis after swimming training (Nordsborg et al. 2015).

Overall, it appears that even a small degree of contractile activity during exercise training results in muscular adaptations in sedentary women. However, it may also be speculated that *m*. vastus lateralis activation during swimming is higher in sedentary women than in trained swimmers (Ikuta et al. 2012) and was thus the cause of the observed adaptations in leg muscle after swimming training.

### Method considerations

In the current study, the relatively high number of investigated proteins, together with the use of several statistical analyses for each protein to evaluate the hypotheses, increases the probability of making statistical type II errors. Some of the results may therefore be discounted as false‐positive findings. By way of example, the expression of Na^+^/K^+^ pump *α*_1_ and GS was increased in *m*. deltoideus in the control group, while levels of the *β*_1_ subunit, FXYD1, NHE1, and SOD1 were elevated solely in vastus lateralis muscle in one of the swimming training groups. However, the long intervention period, together with the relatively high number of observations included in each analysis in the present study in comparison with other comparable studies, reduces the probability of both type I and type II errors, allowing firm general deductions to be made. Moreover, the results obtained from *m*. deltoideus may not represent possible adaptations in other upper‐body muscle groups. The present findings highlight the importance of muscle group‐specific evaluations of the response to exercise training which should be considered in future studies. In this context, it is of importance to consider the method utilized for normalization of the investigated protein expressions. In the present study, expression was normalized to the loaded amount of protein as done previously (Nordsborg et al. 2015), but it may be suggested to use other approaches such as applying staining to quantify the loaded amount of protein (Yonan et al. 2005) or even utilizing normalization to expression of a protein which is unaffected by the investigated conditions, with one possibility being alpha‐tubulin (Morales‐Alamo et al. 2013). However, it is required to demonstrate that a chosen endogenous control is unaffected by the investigated condition which is why normalization to the total amount of protein loaded, as done in the present study, seems like a more robust method.

In conclusion, the capacity for lactate extrusion is lower in arm than in leg muscle, and the adaptive response of the lactate/H^+^ cotransporter to exercise training does not differ between the investigated muscle groups. In contrast, upper‐body musculature, compared to lower‐body musculature, shows greater capacity for Na^+^‐dependent H^+^ extrusion, which may have been the cause of higher adaptive potential in leg muscle. Furthermore, Na^+^ and K^+^ handling capability is lower in arm than in leg muscle, and Na^+^/K^+^ pump *α*_2_ isoform adaptability is higher in arm musculature. However, upper‐ and lower‐ extremity musculature shows similar Na^+^/K^+^ pump *β*_1_ and FXYD1 expression, yet arm muscle shows lower adaptive potential of the *β*_1_ isoform as well as FXYD1 compared to leg muscle. Finally, we demonstrate that expression of GLUT4 and SOD2 increases in upper‐body musculature as a result of intense exercise training.

## Conflict of Interest

No conflict of interests are declared by the authors.
